# University College Hospital

**Published:** 1901-05-18

**Authors:** 


### UNIVERSITY COLLEGE HOSPITAL.

### SPECIAL APPEAL.

In consequence of the lamented decease of her late Majesty, Queen Victoria, patron of the hospital since its foundation in 1833, the committee has decided not to hold the usual festival dinner in aid of the funds of the charity; but they are compelled to issue a Special Appeal to enable them to carry on the hospital in a full state of efficiency.

It is common knowledge that public charities have suffered in consequence of appeals in connection with the war in South Africa and depression in trade, but the sick and suffering in our hospitals have had nevertheless to be cared for, and the consequent expenditure has been more difficult to meet than in prosperous times.

The dividends, annual subscriptions, and other reliable income from all sources being about £8,000, and the necessary expenditure being now over £20,000 per annum, the difficulty the committee have in carrying on the institution will be fully understood. In consequence of the erection of the new hospital, portions of which are now in use, the expenditure has unavoidably much increased, especially under the heads of wages, fuel and lighting. It is estimated that when the whole hospital is completed, the total annual expenditure will be between £28,000 and £30,000, so that it will be seen that the adequate support of the enlarged and modern hospital will necessitate a much larger amount of voluntary assistance.

During the past year 2,696 in-patients, and 37,453 out-patients and casualties have been treated within the walls of the hospital. The attendances made by the out-patients and casualties amounted to the enormous total of 131,086.

We earnestly appeal for contributions from the charitable public, both in London and the country, under one or other of the following heads:—

1. Annual subscriptions, an increase of which is earnestly desired by the committee.
2. Donations to the general funds, to be applied at the discretion of the committee.
3. Donations for investment, thus providing a perpetual increase of the maintenance fund. £2,000 (in one sum or by instalments) will endow a bed or cot, which will be named after, or in accordance with the wishes of the donor.

The committee also venture to urge the claims of the charity upon testators.

BEDFORD, President of Hospital.
MONKSWELL, Treasurer of Hospital.
HENRY LUCAS, Chairman of Hospital Committee.
WALTER BAILY, Vice-Chairman of Hospital Committee.

Donations and subscriptions may be sent to the above, to Messrs. Coutts and Co., 59 Strand, W.C., or to Mr. Newton H. Nixon, Secretary of the Hospital.

